# Efficacy of a 7-day course of furazolidone, levofloxacin, and lansoprazole after failed *Helicobacter pylori *eradication

**DOI:** 10.1186/1471-230X-9-38

**Published:** 2009-05-26

**Authors:** Jaime N Eisig, Fernando M Silva, Ricardo C Barbuti, Tomás Navarro Rodriguez, Peter Malfertheiner, Moraes Joaquim PP Filho, Schlioma Zaterka

**Affiliations:** 1 Serviço de Gastroenterologia Clínica, Hospital das Clínicas da Faculdade de Medicina da Universidade de São Paulo, Av Dr Enéas de Carvalho Aguiar, 155, 9°, Andar, Cerqueira Cezar, São Paulo, SP, Brasil; 2 Clinic of Gastroenterology, Hepatology and Infectology, Otto-von-Guericke University Magdeburg, Magdeburg, Germany

## Abstract

**Background:**

Increasing resistance to clarithromycin and nitroimidazole is the main cause of failure in the *Helicobacter pylori *eradication. The ideal retreatment regimen remains unclear, especially in developing countries, where the infection presents high prevalence and resistance to antibiotics. The study aimed at determining the efficacy, compliance and adverse effects of a regimen that included furazolidone, levofloxacin and lansoprazole in patients with persistent *Helicobacter pylori *infection, who had failed to respond to at least one prior eradication treatment regimen.

**Methods:**

This study included 48 patients with peptic ulcer disease. *Helicobacter pylori *infection was confirmed by a rapid urease test and histological examination of samples obtained from the antrum and corpus during endoscopy. The eradication therapy consisted of a 7-day twice daily oral administration of lansoprazole 30 mg, furazolidone 200 mg and levofloxacin 250 mg. Therapeutic success was confirmed by a negative rapid urease test, histological examination and 14C- urea breath test, performed 12 weeks after treatment completion. The Chi-square method was used for comparisons among eradication rates, previous treatments and previous furazolidone use.

**Results:**

Only one of the 48 patients failed to take all medications, which was due to adverse effects (vomiting). Per-protocol and intention-to-treat eradication rates were 89% (95% CI- 89%–99%) and 88% (88–92%), respectively. Mild and moderate adverse effects were reported by 41 patients (85%). For patients with one previous treatment failure, the eradication rate was 100%. Compared to furazolidone-naïve patients, eradication rates were lower in those who had failed prior furazolidone-containing regimen(s) (74% vs. 100%, p = 0.002).

**Conclusion:**

An empiric salvage-regimen including levofloxacin, furazolidone and lansoprazole is very effective in the eradication of *Helicobacter pylori*, particularly in patients that have failed one prior eradication therapy.

## Background

Consensus meetings on *H. pylori *have advised that the first-line treatment should be a drug regimen that includes a PPI and two antimicrobial agents (clarithromycin + amoxicillin or clarithromycin + metronidazole). The triple therapy usually results in an eradication rate > 80%. A high rate of *H. pylori *primary resistance to nitroimidazole has been observed in developing countries[1]. Resistance to clarithromycin and nitroimidazole is responsible for most *H. pylori *treatment failures [2,3].

In Brazil, primary bacterial resistance to metronidazole and clarithromycin is around 56% [4] and 9% [5] respectively. Whereas the first-line eradication regimens fail to eradicate *H. pylori *in 5 to 20% of patients in developed countries[6], this percentage tends to be higher in developing countries such as Brazil (15–30%) [7-10].

The ideal triple therapy should be effective, safe and low-cost, which is a difficult goal to achieve [11-13].

Furazolidone is a synthetic nitrofuran derivative with bactericidal or bacteriostatic activity when used against Gram-positive and Gram-negative bacteria and it is well absorbed in the intestine with no significant tissue accumulation [14]. It has anti-*H. pylori *activity and furazolidone-resistant strains appear to be rare or non-existent in many areas [15], making this drug a potential option in a rescue regimen for *H. pylori *eradication.

On the other hand, the efficacy of levofloxacin, a levorotatory isomer of ofloxacin with known activity against *H. pylori *[16] has been assessed in several eradication studies [17,18]. Some recent reports have shown promising results for levofloxacin-based rescue regimens in the treatment of persistent *H. pylori *infection. In general, the available clinical trials have involved small numbers of patients and have demonstrated variable eradication rates, ranging from 63% to 100% [19-21]. Therefore, the aim of the present study was to determine the efficacy, compliance rate and adverse effects of a regimen including furazolidone, levofloxacin and lansoprazole in the retreatment of *H. pylori *infection in patients with active or healed duodenal ulcers, who had failed to respond to at least one prior regimen for *H. pylori *eradication.

## Methods

This study was a prospective, open-label trial for the treatment of 48 *H. Pylori*-positive patients with peptic ulcers, who had previously failed one or more eradication regimens (Table 1). The enrolled patients were selected from the Gastroenterology Outpatient Clinic of Hospital das Clinicas, School of Medicine, University of São Paulo (USP). The study was approved by the Ethics Committee of the USP and patients who agreed to participate in the study gave their written informed consent. Bacterial persistence after treatment was confirmed by positive 14C- urea breath test and rapid urease test and histological examination using a modified Giemsa staining method. Gastric mucosa samples were obtained from the antrum and corpus through upper digestive endoscopy.

**Table 1:** Characteristics of population included in study

age (years)	mean	48
	median	48
	range	20 – 71
sex	female	29 (60%)
tobacco use		17 (35%)
ulcer type	duodenal	33 (69%)
	gastric	11 (23%)
	gastric + duodenal	4 (8%)
previous failed treatments	1	28 (58%)
	2	13 (27%)
	3 or more	7 (15%)

Patients who received antisecretory drugs, antibiotic therapy, bismuth compounds or nonsteroidal anti-inflammatory drugs, including low-dose aspirin, up to four weeks before the start of the study were excluded from it. Previous gastrointestinal surgery, pregnancy or lactation and severe comorbidity were the other exclusion criteria.

The patients were submitted to an eradication therapy regimen consisting of a 7-day, twice-daily oral administration of lansoprazole (30 mg), furazolidone (200 mg) and levofloxacin (250 mg). Patients were advised not to drink alcoholic beverages and to avoid foods associated with potential side effects determined for drugs similar to monoamine oxidase inhibitors. They were also encouraged to follow the prescribed regimen regularly and were informed about the importance of the adequate use of the medication for a successful treatment.

No other medication was allowed until the end of the treatment, when patients were evaluated regarding compliance by counting the remaining tablets. Therapeutic compliance was defined as the intake of 100% of the prescribed medication. Adverse events were recorded in a questionnaire, and each clinical adverse effect was carefully investigated.

Treatment efficacy was determined by bacterial negativity in at least two of three diagnostic methods: rapid urease test, histological examination of gastric antrum and corpus mucosa samples or 14C- urea breath test, performed 12 weeks after completion of the eradication treatment.

### Statistical Analysis

Eradication rates were analyzed on an intention-to-treat and per protocol basis. A confidence interval of 95% was calculated for the eradication rate percentiles. The Chi-square method with Pearson's coefficient was used for comparisons among the variables, eradication rates, number of previous treatment, previous use of furazolidone, and statistical significance was set at p < 0.05.

## Results

Forty-eight patients, of which 29 (60%) were females, with a mean age of 48 years (range: 20–71 years) were included in this study: five presented with active ulcers, while ulcer scars were observed in 43 of the patients. Twenty-eight patients had experienced one previous treatment failure (Table 1). The contents of the previous anti-*H. pylori *therapies are shown in Table 2.

**Table 2:** Composition of Previous Treatment

	previous treatment	number of patients
**1×**	OAC	18
	OAA'	7
	OFT	2
	OMC	1

subtotal		**28**

**2×**	OAA' + OFT	5
	OAC + OFT	4
	OAA' + OAC	2
	OAC + OMC	2

subtotal		**13**

**3 × or more**	OAA' + OAC + BAFO	2
	OAC + OFT + BAFO	2
	OMC + OAC + BAMO	1
	OAA' + OAC + OFT + BAFO	1
	OMC + OAC + OFT + BAFO	1

subtotal		**7**
total		**48**

Legend: O: omeprazole, A: amoxicillin, A': azithromycin, F: furazolidone, T: tetracycline, M: metronidazole, C: clarithromycin, B: subcitrate-bismuth.

All but one of the patients took 100% of their prescribed medication. This patient experienced significant vomiting and was excluded from the per-protocol analysis. Mild and moderate adverse effects were reported by 41 patients (85%). The most frequent complaint was nausea, reported by 37% of the patients.

Eradication rate was respectively 89% (95% CI: 89–99%) and 88% (95% CI: 88–92%) in per protocol and intention-to-treat analyses. Treatment success was related to the number of previous treatments and to the previous use of furazolidone (Table 3). All patients previously submitted to only one treatment achieved successful eradication, but when 3 or more previous treatment regimens had failed, the eradication rate decreased to 57% (p = 0.004). All patients with no previous use of furazolidone achieved *H. pylori *eradication.

**Table 3:** Per protocol Eradication Rates, Previous Failures and Furazolidone previous use

previous failures	eradication rates	eradication rates with furazolidone previous use	eradication rates without furazolidone previous use	p
*1	(28/28) 100%	(2/2) 100%	(26/26) 100%	
2	(10/17) 83%	(7/9) 78%	(3/3) 100%	0.68
*3 or more	(4/7) 57%	(3/6) 50%	(1/1) 100%	0.35

Total	(42/47) 89%	(14/19) 74%	(28/28)100%	0.002

*p = 0.004

## Discussion

First-line regimens for *H. pylori *treatment in Brazil usually include a seven-day course of PPI, clarithromycin and amoxicillin. The negative impact of clarithromycin resistance on the therapy success for *H. pylori *eradication is remarkable, decreasing the eradication rate by more than 20% [22].

International guidelines and the Brazilian consensus recommend the quadruple therapy with PPI, bismuth subcitrate, tetracycline, and metronidazole as a second-line therapy in case of treatment failure [1,23,24]. The disadvantages of this bismuth-based quadruple therapy include the high daily pill count and frequent side effects, which affect treatment compliance, as well as the high *H. pylori *resistance to nitroimidazole compounds observed in our country.

In the past years, new antibiotics have emerged as potential agents against antibiotic-resistant *H. pylori *strains, such as rifabutin and levofloxacin. Rifabutin provides eradication rates that range from 79% to 90%, when combined with amoxicillin and PPI [25,26], but it is a very expensive drug, it is has been associated with myelotoxicity and it is not commercially available in Brazil.

A recent meta-analysis showed that the levofloxacin-based triple therapy with amoxicillin plus PPI offers a number of advantages over the bismuth-based quadruple therapy, such as better overall tolerance and higher efficacy [27].

Furazolidone provides an excellent alternative to the combined therapy for *H. pylori *eradication [20,28,29], in substitution to amoxicillin or metronidazole, resulting in good eradication rates in patients who have had a failed treatment; however, its use has not become widespread due to the reported side effects.

The present study confirms the results of Gonzaga et al study [20], in which eradication rate was 100% (PP analysis). Unfortunately the number of patients in the study was very small (12 patients, 2 lost to follow-up). According to this study, the necessary sample size to define the statistical difference for an eradication rate of 85–90% would be of at least 40 patients. Our study included 48 patients. The overall treatment success was 89% (42/47) per protocol and when patients had previously received only one treatment, eradication success was 100%.

Our results are similar to those reported by Nista et al [19] (PPI+ levofloxacin + tinidazol or amoxicillin) and Sharara et al [30] (PPI + gatifloxacin and amoxicillin), although treatment regimens were different. It not advisable to include nitroimidazole in eradication regimens in Brazil, as primary resistance to this antibiotic agent is high [1,4,5] and gatifloxacin is not commercially available in Brazil.

Amoxicillin, an inexpensive drug [31], has been widely used in first-line and rescue *H. pylori *eradication regimens. At least one study in Brazil [32] has shown a very high resistance to amoxicillin (38%), thus making the inclusion of amoxicillin in retreatment regimens a difficult decision to be made.

The present study confirms that the rescue therapy efficacy is directly related to the number of previous treatments. In patients previously submitted to 3 or more treatments, the eradication rate was 57% (4 of 7) and among those previously submitted to one treatment, it was 100% (28 of 28). Of the 28 patients submitted to one previous treatment, 26 had not received furazolidone. When furazolidone had been previously included in the treatment regimen, the eradication success decreased significantly (p = 0.002).

The duration of treatments that include levofloxacin is another question for debate. Although a seven-day eradication regimen was effective [28,33] an increase in the eradication rate was observed when a ten-day regimen was prescribed. The advantage of a seven-day regimen is the high adherence, lower cost and fewer adverse events.

Side effects reported with levofloxacin-based regimens plus amoxicillin are usually rare. It is possible that most of the side effects reported by our patients were furazolidone-related. Nevertheless, contrary to what usually is reported by others [34-36], side effects observed in our study were usually mild or moderate, well tolerated and did not interfere with patients' compliance.

Empirical rescue regimens used for *H. pylori *eradication treatment are a matter of discussion. Although recent guidelines suggest microbial sensitive tests for *H. pylori *retreatment, bacterial cultures are expensive, time-consuming and unavailable as routine tests, particularly in most of the developing countries.

In Brazil, the rescue regimen suggested in this study has a low cost (US$ 26.00), consisting of only three capsules bid [37-39], being adequately supplied by the public health services.

## Conclusion

Our results demonstrate that the empiric salvage-regimen for *H. pylori *eradication including levofloxacin, furazolidone and PPI is very effective, particularly in patients previously submitted to a maximum of 2 treatment regimens.

## Competing interests

The authors declare that they have no competing interests.

## Authors' contributions

JNE participated in the acquisition of data and in quality control, participated in analysis and interpretation of data and drafted the manuscript. FMS and TNR participated in the acquisition of data, in the quality control and in analysis and interpretation of data. PM, JPPMFo., and SZ participated in analysis and interpretation of data. RCB performed the endoscopic examinations. All authors contributed to the design of the study, read and approved the final manuscript.

## Pre-publication history

The pre-publication history for this paper can be accessed here:

http://www.biomedcentral.com/1471-230X/9/38/prepub
